# Loss of Melanopsin Photoreception and Antagonism of the Histamine H3 Receptor by Ciproxifan Inhibit Light-Induced Sleep in Mice

**DOI:** 10.1371/journal.pone.0128175

**Published:** 2015-06-17

**Authors:** Fanuel Muindi, Damien Colas, Jesse Ikeme, Norman F. Ruby, H. Craig Heller

**Affiliations:** 1 Department of Biology, Stanford University, Stanford, California, United States of America; 2 Department of Brain and Cognitive Sciences, Massachusetts Institute of Technology, Cambridge, Massachusetts, United States of America; University of Lübeck, GERMANY

## Abstract

Light has direct effects on sleep and wakefulness causing arousal in diurnal animals and sleep in nocturnal animals. In the present study, we assessed the modulation of light-induced sleep by melanopsin and the histaminergic system by exposing mice to millisecond light flashes and continuous light respectively. First, we show that the induction of sleep by millisecond light flashes is dose dependent as a function of light flash number. We found that exposure to 60 flashes of light occurring once every 60 seconds for 1-h (120-ms of total light over an hour) induced a similar amount of sleep as a continuous bright light pulse. Secondly, the induction of sleep by millisecond light flashes was attenuated in the absence of melanopsin when animals were presented with flashes occurring every 60 seconds over a 3-h period beginning at ZT13. Lastly, the acute administration of a histamine H3 autoreceptor antagonist, ciproxifan, blocked the induction of sleep by a 1-h continuous light pulse during the dark period. Ciproxifan caused a decrease in NREMS delta power and an increase in theta activity during both sleep and wake periods respectively. The data suggest that some form of temporal integration occurs in response to millisecond light flashes, and that this process requires melanopsin photoreception. Furthermore, the pharmacological data suggest that the increase of histaminergic neurotransmission is sufficient to attenuate the light-induced sleep response during the dark period.

## Introduction

Light serves as an important regulator of behavior and physiology in mammals. A key example of this is the circadian system which is entrained by light and, in turn, influences the timing of sleep and wake, locomotor activity, and hormone production [1]. In nocturnal animals, a light pulse during the dark phase suppresses locomotor activity (negative masking) and rapidly induces sleep in both a phase dependent and dose-dependent manner [2–4]. In mammals, the eye serves as the only input of photic information into the brain [5]. The rod, cone, and melanopsin photoreceptors in the eye give mammals the ability to perform both image and non-image forming functions ranging from the pupillary light reflex to sleep induction in mice [6–11].

Studies to date have primarily focused on the contribution of rod, cone, and melanopsin photoreception in the acute induction of sleep by using continuous light pulses [3,11–13]. We and others have previously shown that the circadian system is sensitive enough to respond to millisecond light flashes to produce large phase shifts and the suppression of locomotor activity [14–17]. Using mostly locomotor activity, recent studies have suggested that millisecond light flashes may be sufficient to induce sleep in mice [18–20]. The contribution of the different photoreceptors to the millisecond flash-induced sleep is not fully clear. Equally important in the acute induction of sleep by light is the question about the involvement of areas innervated by the retino-hypothalamic tract (RHT). Photic information from retinal photoreceptors reaches a broad range of retino-recipient areas in the brain via the RHT [21]. In the hypothalamus, the RHT innervates a number of areas involved in the regulation of sleep and wake. These areas include the suprachiasmatic nucleus (SCN), subparaventricular zone (SPZ), ventral lateral preoptic area (VLPO), and the lateral hypothalamus (LH). Although these projections are relatively well characterized, the involvement of these areas during the light-mediated transition from wake to sleep is not well understood. One hypothesis is that light directly impinges on the sleep-wake system activating the sleep-active neurons in the ventrolateral preoptic area (VLPO) thus causing the shift toward sleep induction via the inhibition of the wake-promoting areas in the brain [22,23].

One area whose involvement in light-induced sleep has not been studied is the tuberomammillary nucleus (TMN). Located in the posterior hypothalamus, the TMN contains histamine producing neurons [24,25]. The histaminergic neurons in the TMN innervate a large number of brain areas [26], are more active during wake [27] and are part of the wake-promoting system which include, the dorsal raphe nucleus (DR), locus coeruleus (LC), and the hypocretin (HCRT) neurons in the lateral hypothalamus [28]. An important feature of the histaminergic system is that histamine inhibits its own release and synthesis via the histamine H3 pre-synaptic autoreceptor [29,30]. As such, administration of the histamine H3 receptor antagonists increase the synthesis and release of histamine [29,31–33] resulting in an enhancement of wakefulness and cortical theta rhythms [34–37].

In the present study, we have (i) investigated the acute effects of millisecond light flashes on sleep and wake to better understand the integrative capacity of the mouse photic system, (ii) assessed the contribution of melanopsin photoreception in the acute effects of light flashes on sleep, and (iii) examined whether an increase in histaminergic neurotransmission via ciproxifan, a selective histamine H3 receptor antagonist [38], is sufficient to prevent the acute induction of sleep by light early in the dark period.

## Materials and Methods

### Animals

Adult, 3–4 month old C57BL/6J male wildtype (WT) and melanopsin knockout (MKO) mice (backcrossed eight generations to the C57BL/6J background in our lab) were used [3]. Mice were housed individually in plastic cages with *ad libitum* access to food and water. The animals were housed in 12:12 LD light cycle in a room that contained four cool white fluorescent tubes (34 W; GE Lighting, Pleasanton CA) producing an intensity of approximately 40 μW/cm^2^ on the cage floors. All animal experiments were carried out in accordance with the National Institutes of Health Guide for the Care and Use of Laboratory Animals and approved by Stanford University Administrative Panel on Laboratory Animal Care.

### Light stimulation

Millisecond Light Flashes for Experiments 1 and 2: 2-ms light flashes were generated by a broad emission tungsten lamp (~200 μW/cm^2^) directed toward the center of the animal cage rack approximately 0.5 m away. The lamp was controlled by the Master-8 Pulse Stimulator (A.M.P.I., Jerusalem, Israel) which was programmed via the Master-8 Control Software. Continuous Light Pulse for Experiment 3: A separate white, dimmable LED lamps (LumaPro A19 8W; Grainger, San Jose CA) positioned above each individual cage were used for continuous light pulses. The intensity was controlled by a variable autotransformer (Model 116B, Superior Electric Company, Bristol CT) and measured by using an IL-1400 SL021 #385 photodetector (International Light, Newburyport, MA) as previously described [3].

### Surgery and sleep data acquisition

A Somnologica system (Embla, Denver CO) was used to acquire the cortical electroencephalographic (EEG) and electromyographic (EMG) signals. Implantation of EEG/EMG electrodes was done as previously described [3,39]. EEG electrodes were placed over the frontal and parietal cortex. 14 days were allowed for recovery after surgery. Animals were connected to lightweight recording cables and adapted for at least 5 days before experiments began. All signals were amplified, analog-to-digital converted with a sampling rate of 200 Hz, and digitally filtered (EEG, 0.5–70 Hz; EMG, 10–70 Hz) as previously described [39]. Behavioral states of the animals were scored in 4-s epochs using visual inspection of EEG and EMG signals for non-rapid eye movement sleep (NREMS; high voltage, low frequency EEG), rapid eye movement sleep (REMS; prominent theta activity, low voltage EMG), and wake (low voltage, high frequency EEG) [3]. The power spectra of the corresponding EEG signals was calculated by using fast Fourier transformation (sampling at 256 Hz) for 4-s epochs. Power in the 0.8–40 Hz range of the recording was averaged, and the mean values were plotted in 0.8 Hz bins. Ratio of power in delta (δ) range and theta (θ) range were calculated by using the aggregate power between 0.8–4 Hz and 4.8–8 Hz respectively.

### Protocols

#### Experiment 1

**Fig 1:**
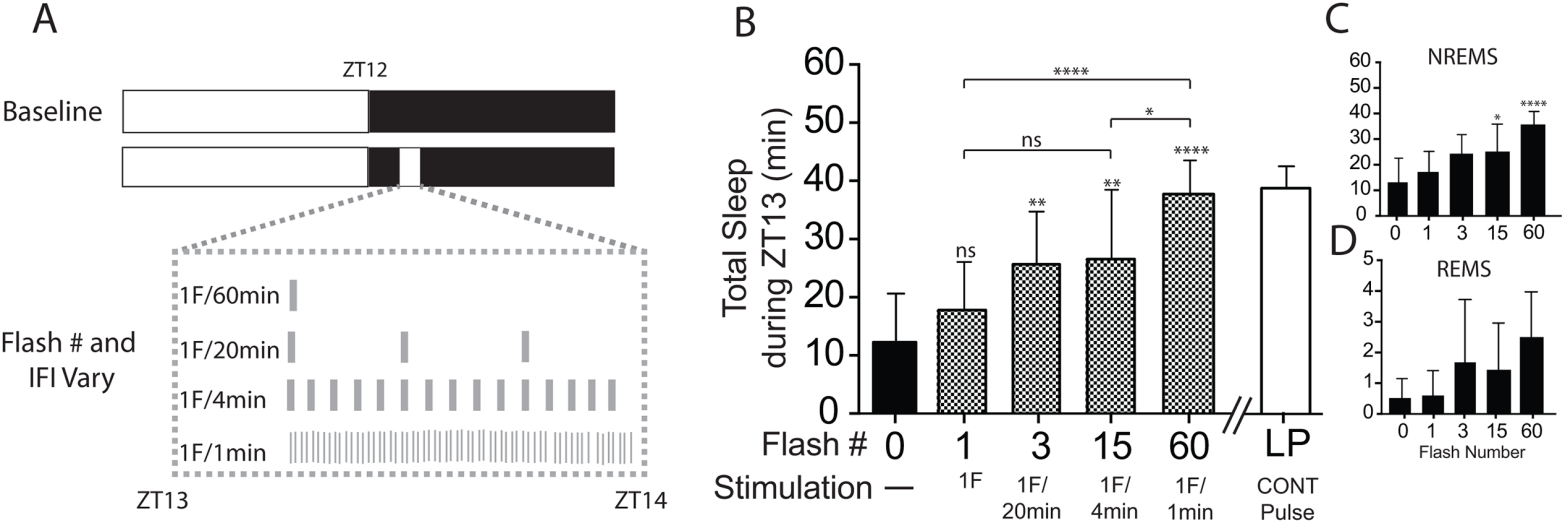
Sleep induction in response to different number of light flashes. (A) Wildtype mice (n = 8) received equally distributed light flashes (0, 1, 3, 15 and 60) across a 60 minute period beginning at ZT13. (B) Total sleep (NREMS+REMS), (C) NREMS, and (D) REMS are shown. *p < 0.05, **p < 0.01 ****p < 0.0001; versus baseline (0 pulses) by one-way repeated measures ANOVA followed by Bonferroni's *post-hoc* test. The continuous pulse data (white bar) are from Muindi et al. (2013). Data expressed as mean ± SD.

We first assessed the ability of millisecond light pulses to induce sleep. One group of WT mice (n = 8) was pulsed with light flashes (0, 1, 3, 15 and 60) evenly over an hour on different days (Fig 1A). Light flashes were initiated 1-h (ZT13) after lights-off, and the order of light flash delivery was randomized. There was a minimum of 3 days between pulse protocols to allow for adequate recovery after pulses.

#### Experiment 2

We assessed the contribution of melanopsin during photosomnolence in response to millisecond light flashes. A new group of WT (n = 8) and MKO (n = 6) mice were administered flashes at 60 second intervals over a 3-h-period beginning at ZT13. The 3-h period was chosen because previous studies have shown that this time window is necessary to fully assess the ability of melanopsin for masking and sleep [3,10,12].

#### Experiment 3

To test the effects of ciproxifan on the acute induction of sleep, a group of mice (n = 7) was injected intraperitoneally with either 0.9% saline or ciproxifan (12mg/kg) 20 min before exposure to a continuous light (20 μW/cm²) for one hour beginning at ZT13. In order to better observe the animals during the experiment, we chose to use a continuous 20 μW/cm² light pulse. It is important to note that the continuous light pulse we chose induced similar amounts of sleep as exposure to 60 flashes of light over a 1-h period (Fig 1B). The drug, ciproxifan, was dissolved in sterile saline. Each animal was tested 4 times using the following protocol: a) injection of saline with no light exposure; b) injection of ciproxifan with no light exposure; c) injection of saline with light exposure; d) injection of ciproxifan with light exposure. The order of the conditions was counterbalanced across animals over the 4 conditions. A minimum of 3 days was used to separate the conditions. This period of time was chosen because we have previously shown that it is sufficient for recovery from a light pulse [3]. Furthermore, the chosen period of time was also sufficient for recovery in animals injected with ciproxifan since the drug has been shown to have a short lasting waking effect and does not affect total sleep amounts [37].

### Statistical analysis

GraphPad Prism (GraphPad Software, Inc. Version 6.0a) was used for all statistical calculations. One-way or two way repeated measures ANOVAs were performed on data that satisfied assumptions of normality and variance homogeneity through the Brown-Forsythe test followed by Bonferonni’s or Tukey’s test for *post hoc* comparisons. Differences in the power of frequency bands (delta and theta) between control (saline) and drug (ciproxifan) were analyzed by paired Student’s *t* test. All data are expressed as mean ± SD.

## Results

### The influence of light flash number and interval on total sleep induced early in the dark period

We first assessed the ability of millisecond light flashes to induce sleep early in the dark period. A one-way repeated measures ANOVA revealed a significant effect of flash number on total sleep (*F*_(4,28)_ = 18.5; *p* < 0.0001) and NREMS (*F*_(4,28)_ = 11.0; *p* < 0.0001). However, no effect of flash number was detected on REMS (*F*_(4,28)_ = 2.59; *p* = 0.056). The one flash stimulus failed to induce significant changes in total sleep whereas the 60 flash stimulus was able to induce the maximum amount of sleep (25.4 ± 1.79 min above baseline; p < 0.0001; *post hoc t test*) (Fig 1B). A similar response was observed for NREMS (Fig 1C). Although an increase in REMS was detected, post hoc analyses failed to identify significant differences between baseline and the different flash number paradigms (Fig 1D). An increase of only ~2 min of REMS was observed in response to the 60 flash stimulus. As such, all subsequent analyses of sleep amounts are summations of both NREMS and REMS (i.e., total sleep). The majority of sleep induction occurred in the first 30 min across all the different flash paradigms (S1A–S1E Fig). Additionally, the data show that exposure to 60 millisecond light flashes delivered over a 2 minute period is sufficient to induce sleep post flash stimulation (S2 Fig).

### Sleep response to light flashes in the absence of melanopsin

**Fig 2:**
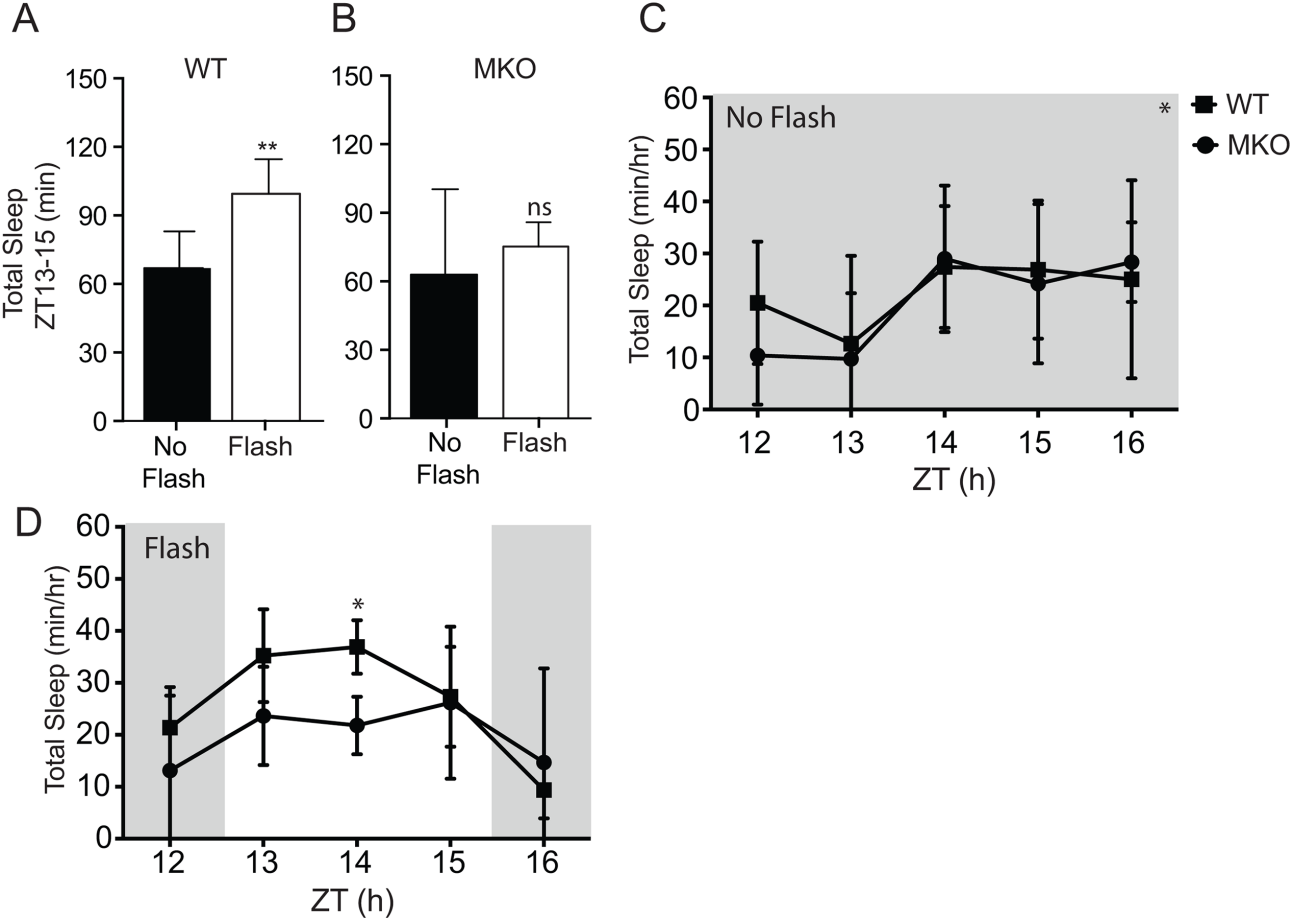
Melanopsin required for millisecond light flash induced sleep early in the dark period. (A,B) Total sleep (NREMS +REMS) during flash stimulation delivered once every 60 seconds for 3 hours between ZT13-15 is shown in WT (n = 8) and MKO (n = 6) mice. Baseline period is shown by the black bars (NO FLASH) and the stimulation period is shown by the white bars (Flash ON). **p = 0.009, paired *t* test. (C,D) Hourly total sleep between ZT12-16 is shown for WT and MKO mice during baseline (top graph) and stimulation (bottom graph). During baseline, no effect of genotype was detected. However, an effect of time (*) was detected. During the flash stimulation (ZT13-15), two-way repeated measures ANOVA reveals an effect of genotype but no time effect. No interaction was detected (see text for details). *p < 0.05 by Bonferroni’s *post hoc t* test versus WT mice. Data are expressed as mean ± SD.

A significant induction of sleep above baseline (~49%) was observed in WT mice (n = 8) in response to light flashes separated by 60 seconds over the 3 h-period (66.9 ± 16.1 min during baseline versus 99.5 ± 15.2 min during flashes; *p* = 0.009, paired *t test*). An increase (~20%) was observed but did not reach statistical significance in MKO mice (n = 6) (62.9 ± 37.5 min during baseline versus 75.3 ± 10.6 hrs during flashes; *p* = 0.47, paired *t test*). We next examined the temporal extent to which total sleep amounts changed between ZT13-15 in WT and MKO mice by using two-way repeated measures ANOVA (Fig 2C and 2D). During baseline, no effect of genotype was detected (factor ‘genotype (*F*_(1,12)_ = 0.08; *p* = 0.79). However, an effect of time was detected (*F*_(2,24)_ = 6.82; *p* = 0.0045). No interaction was detected (*F*_(2,24)_ = 0.13; *p* = 0.88). During light flash exposure, genotype effect was detected (factor ‘genotype (*F*_(1,12)_ = 11.1; *p* = 0.006). However, no effect of time was detected (*F*_(2,24)_ = 0.37; *p* = 0.70) nor was there an interaction (*F*_(2,24)_ = 2.07; *p* = 0.15). These results suggest that melanopsin photoreception is required to mediate the full somnogenic effects of light flashes early in the dark period.

### Effects of ciproxifan on the acute induction of sleep early in the dark period

**Fig 3:**
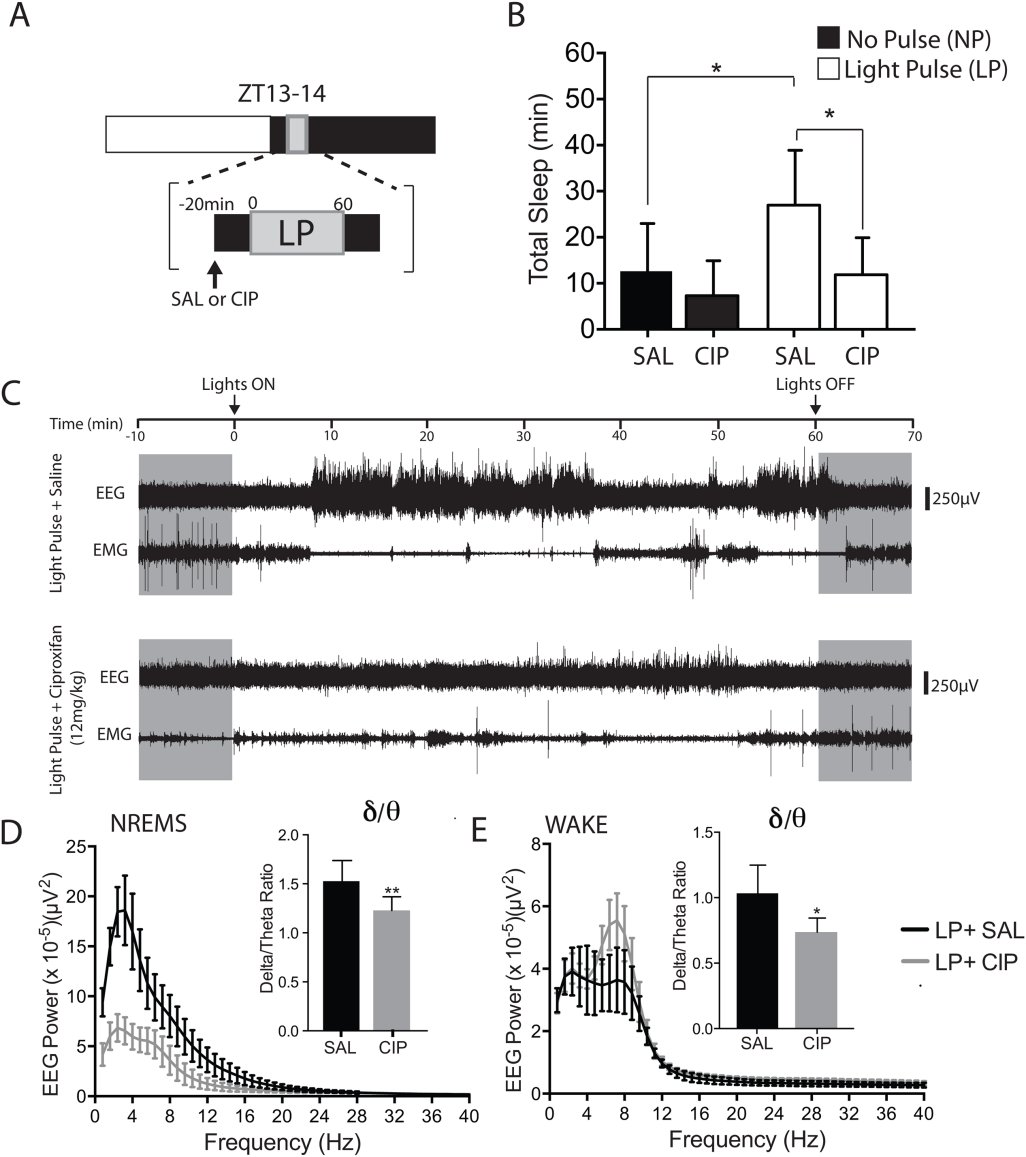
Effects of ciproxifan administration on light-induced sleep and cortical EEG early in the dark period. (A) An illustration of the protocol used to assess the effect of ciproxifan administration on light-induced sleep is shown. Twenty minutes before exposure no light or continuous light at ZT13, mice (n = 7) were administered with saline (SAL) or ciproxifan (CIP) (12mg/kg, i.p). The light exposure lasted one hour. (B) Total sleep in response to saline or ciproxifan during the no pulse (NP) condition and the light pulse (LP) condition is shown. *p<0.05; by Tukey’s *post hoc t* test after one-way repeated measures ANOVA. (C) Representative EEG and EMG traces are shown after saline and ciproxifan administration in the light condition. (D, E) Power in the 0.8–40 Hz range for artifact-free recordings was averaged, and the mean values plotted as previously described [3]. (*Insets*) Delta/theta ratios during NREMS and wake were calculated and plotted. Data represent mean ± SD (n = 5). *p<0.05, **p<0.001 by paired *t* test.

**Fig 4:**
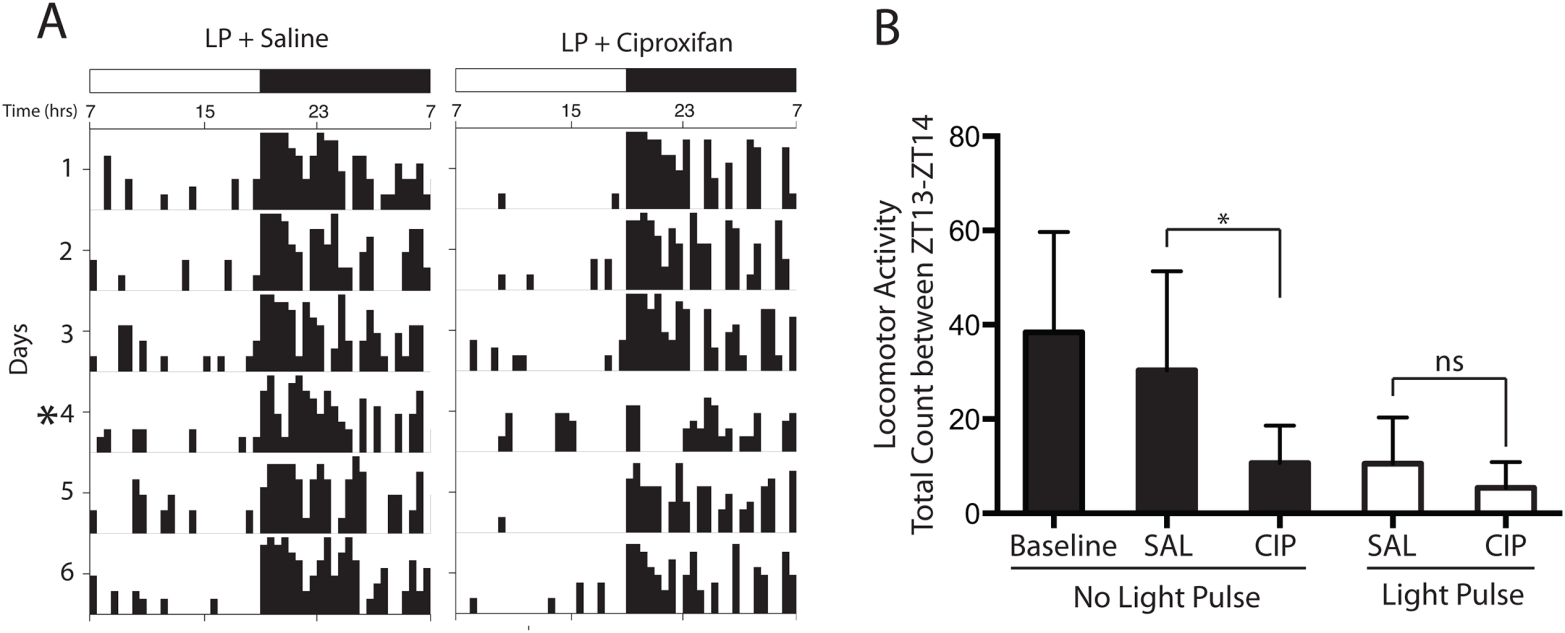
Effects of ciproxifan administration on locomotor activity. (A) Representative actograms from mice exposed to light with saline or ciproxifan. Days are indicated on the left and time during the day is indicated on the top. Light period was between 0700–1900 hrs and the dark period was between 1900–0700 hrs. The asterisk (day 4) indicates the period when the light pulse was given (2000–2100 hrs) after saline or ciproxifan administration 20 min before the pulse. (B) Quantification of locomotor activity (total count between 2000–2100 hrs) during no light pulse (black bars) and light pulse condition (white bars) after saline and ciproxifan administration is shown. Note that baseline refers to no light pulse or administration of either saline or ciproxifan. Data represent mean ± SD (n = 3–7). *p<0.05 by Tukey’s *post hoc t* test after one-way ANOVA for saline and ciproxifan treatments.

The histaminergic system is known to modulate wakefulness via the action of histamine on histamine receptors (H1-4) found in several areas across the brain. The blockade of H3 receptors promotes wakefulness [34–37]. As such, we questioned whether the blockade of H3 receptors by ciproxifan, an H3-receptor antagonist, would be sufficient to block the sleep inducing effects of light in the dark period in WT mice. To test this, mice (n = 7) were injected intraperitoneally with either 0.9% saline or ciproxifan (12mg/kg) 20-min before exposure to a continuous light (20 μW/cm²) for one hour beginning at ZT13. A one-way repeated measures ANOVA revealed a significant effect of ciproxifan treatment on the ability of light to induce sleep (*F*_(3,18)_ = 5.93; *p* = 0.0054) (Fig 3B). No significant difference in total sleep amount was observed between saline and ciproxifan treatments in the no light condition (Fig 3B) (*p* = 0.71; *post hoc t test*). However, a significant increase in total sleep to 27.0 ± 11.9 min was observed when the mice were exposed to light after saline treatment (*p* = 0.04; *post hoc t test*). Ciproxifan blocked this increase in sleep in response to light reducing total sleep to 11.9 ± 8.06 min (*p* = 0.03; *post hoc t test*). No significant differences were observed when comparing ciproxifan treatments between the ‘no-pulse’ and ‘light-pulse’ conditions (*p* = 0.78; *post hoc t test*). We next assessed the quality of sleep and wake by using power spectra analysis on NREMS and wake in the light condition. A representative EEG/EMG trace illustrates the temporal profile of changes in sleep during the light pulse condition after saline and ciproxifan treatments (Fig 3C). There was a significant decrease in the delta/theta ratios during both NREMS (*p* = 0.006, paired Student’s *t* test) and wake (*p* = 0.019, paired Student’s *t* test) after ciproxifan treatment (Fig 3D and 3E) indicating an increase in cortical activity. Additionally, a one-way ANOVA revealed a significant effect of ciproxifan treatment on locomotor activity (*F*_(3,20)_ = 4.41; *p* = 0.0156) (Fig 4B). A decrease in locomotor activity was observed between saline and ciproxifan treatments in the no light condition (*p* = 0.04; *post hoc t test*). However, no differences were observed between saline and ciproxifan treatments in the light condition (*p* = 0.77; *post hoc t test*).

REMS power spectra was not assessed because no REMS was observed in most of the animals after ciproxifan administration (data not shown). Consistent with a previous report [37], total sleep amounts post light pulse (ZT14-24) did not differ between saline and ciproxifan treated animals (5.23 ± 2.57 h after saline vs. 5.27 ± 2.20 h). Using a separate group of mice (n = 4), we also determined the effects of lower ciproxifan doses on the acute induction of sleep by light. Ciproxifan similarly attenuated the sleep inducing effect of light. A reduction of ~16% and ~35% in total sleep during a light pulse was observed at 3mg/kg and 6mg/kg doses respectively (S3 Fig).

## Discussion

### Melanopsin photoreception required for millisecond light flashes to induce sleep

The effects of millisecond light flashes on phase shifts and masking are well documented in several studies [14–17,19]. Experiments using hamsters have shown that as few as three flashes delivered over a 5 or 60-min window are sufficient to elicit large phase advances [17]. Remarkably, only ten flashes similarly distributed over a 5 min period have been reported to induce robust locomotor activity suppression in mice lasting almost 20-min [19]. We extend the findings by showing that as few as three flashes (a total light duration of 6-ms) was sufficient to induce a significant increase in sleep from baseline (Fig 1). Total sleep increased in a dose dependent manner as a function of light flash number suggesting that some form of integration process is taking place to sum the total light exposure in a given time window. We also show that exposure to 60 light flashes delivered over 2 minutes is sufficient to induce sleep for almost 20-min without additional flash exposure (S2 Fig). This result confirms a recent result reported by Studholme et al. (2013) [18] whereby a presentation of 10 light flashes delivered over a 5-minute period similarly induced sleep post exposure. It is likely light flashes initiate a sequence of activation and/or inhibition across both sleep and wake areas resulting in the acute induction of sleep [22]. In the absence of additional light exposure, the response cannot be maintained hence the reduction in sleep for the remaining 15 min intervals (S2 Fig).

We also assessed whether melanopsin photoreception is required during the induction of sleep by millisecond light flashes early in the dark period. Our results show that the somnogenic effects of millisecond light flashes across a 3-hour period are attenuated in the absence of melanopsin (Fig 2). This indicates that melanopsin photoreception plays an important role during the integration of millisecond light flash information before conveying the signal to sleep/wake centers in the brain. This is consistent with results from several studies assessing the role of melanopsin in the induction of sleep by continuous light [3,9,11]. Although higher sleep amounts were observed in WT mice in comparison to MKO mice, no differences in the temporal profiles of sleep were detected across the flash exposure (Fig 2D). This result contrasts with Altimus et al. (2008) and Muindi et al. (2013) where sleep was initiated but not maintained in mice lacking melanopsin during exposure to photopic continuous light. The observation here suggests that the rod/cone photoreceptors alone are unable to compensate for the absence of melanopsin during exposure to millisecond light flashes. It is possible that the absence of melanopsin prevents the integration of light that is known to occur at the level of the retinal ganglion cells involved in non-image forming functions [40]. Interestingly, a recent study showed that the selective activation of ultraviolet-sensitive cones by UV light exposure is sufficient to induce sleep with equal efficacy in both WT and mice lacking melanopsin [13]. This result suggests that the UV light detection in the retina is sufficient to induce and maintain sleep independent of melanopsin. Clearly, additional experiments are required to further assess how changes in the wavelength, intensity and distribution of light pulses affect the induction and maintenance of sleep in the absence of melanopsin in mice. Altogether, our results are consistent with the view that the melanopsin expressing retinal ganglion cells in the retina provide a critical input for mediating non-visual photic information.

The results from millisecond light flash experiments raise an important question regarding both the retinal and brain mechanisms involved in the response. Even though the integration mechanisms cannot be fully inferred from our data, it is clear that the mouse photic system is able to integrate light flashes separated in time and melanopsin plays an important role in this integration. Using a wider range of flash intensities, shorter intervals, and exposure time windows across different circadian phases will be important in future studies in determining the full integrative capacity and the mechanisms involved in the mouse photic pathway.

### Ciproxifan attenuates light-induced sleep early in the dark period

The flip-flop switch model has been used extensively to highlight the control of behavioral state by the sleep and wake-promoting systems [28]. According to this model, sleep-promoting neurons inhibit wake-promoting neurons at sleep onset and during sleep. It was recently hypothesized that light alters the balance of the flip-flop switch by the activation of the sleep-promoting neurons in the VLPO which then inhibit the wake-promoting areas thus promoting sleep [23]. Here, we show that the increase in histaminergic tone via the H3-receptor antagonist [38] is sufficient to attenuate the acute induction of sleep by light early in the dark period (Fig 3B). A similar arousal effect of ciproxifan was also observed at lower dosages (S3 Fig). We also observed a decrease in delta power with a concomitant increase in theta power in response to ciproxifan (Fig 3D and 3E). These results suggest that the cortical activation induced via the inhibition of the H3-receptor by ciproxifan is able to oppose the active induction of sleep by light early in the dark period. This is consistent with results from other studies showing that the inhibition of the H3-receptor is sufficient to promote wakefulness [34–37]. As expected, no significant changes in total sleep amount were observed during the rest of the dark period post-injection. This is consistent with results from previous studies showing that the waking effect of H3-receptor antagonists, thioperamide (10-100mg/kg, i.p) and ciproxifan (1-10mg/kg, i.p) is relatively short lasting with animals returning to baseline sleep amounts between 3–4 hours at the highest dosages used [37].

However, a few questions arise regarding the attenuation of light-induced sleep by ciproxifan via the H3-receptors. (1) What other systems are influenced by ciproxifan’s action on the H3 receptors? (2) What is the effect of ciproxifan on histaminergic neurons in the TMN? The first question is important because in addition to its function as an autoreceptor, the H3-receptor also acts as a hetero-receptor regulating the release of several neurotransmitters including acetylcholine, dopamine, serotonin, and norepinephrine [41]. The suggestion then is that the waking effect mediated by ciproxifan may involve multiple neurotransmitters. Although the extent of involvement of each neurotransmitter in the induction of arousal by ciproxifan is not clear, the modulation of histaminergic neurotransmission by H3-receptor antagonists has been documented in a number of studies. H3-receptor antagonists such as thioperamide or ciproxifan enhance histamine synthesis (Arrang et al., 1987; Ligneau et al., 1998) and release (Itoh et al., 1991; Morrisset et al, 2000). Interestingly, H3-receptor-knockout mice have normal levels of cortical dopamine, norepinephrine and serotonin [42]. Surprisingly, decreased levels of histamine were observed in the cortex which suggested to the authors that a compensatory decrease in histamine may have occurred during development in H3-receptor knockout mice [42]. Consistent with the selective role of H3-receptor in histamine release and synthesis, H3-receptor knockout mice are insensitive to the wake inducing effect of H3-receptor antagonists thioperamide [42] and ciproxifan [43].

Concerning the second question, dense c-fos labeling has been observed in TMN neurons following ciproxifan injection in cats [44]. However, single- and multi-unit recordings are necessary to characterize the temporal profile of TMN neuron activity in response to ciproxifan and light pulses. As expected, the wide projections of the TMN across the brain give the histaminergic system robust control over several areas regulating sleep and wakefulness. With respect to the acute effects of light on sleep, it is likely that the neurons in the TMN are rapidly suppressed during the initiation of sleep in response to light. Presumably, rapid changes in neuronal activity occur in other critical areas involved in the modulation of sleep and wakefulness. Conducting simultaneous recordings in both sleep and wake areas will be crucial in better understanding the changes taking place during the active induction of sleep by light [22]. Taken together, the rapid induction of sleep by light presents a unique opportunity to better understand the neuronal circuits driving the rapid switch from wake to sleep.

### Summary and Conclusions

The present experiments expand on previous studies by showing that mice are able to respond to light flashes over a fairly large range of intervals (Fig 1). Additional evidence suggests that the sleep induction in response to millisecond flashes during the early dark period is melanopsin dependent (Fig 2). Lastly, the data also suggest that an increase in histaminergic tone is sufficient to attenuate the increase in sleep by light early in the dark period (Fig 3). Altogether, the findings highlight the sensitivity and integrative capacity of the mouse photic system to light, and suggest that both retinal and downstream brain areas play important roles in processing photic information during the induction of sleep early in the dark period.

## Supporting Information

S1 Fig Temporal profiles of total sleep across different light flash numbers. Total sleep time is shown for control (A) and light flashes (B-E) in 30-min bins before during and after the flashes. Data represent mean ± SD. A one-way repeated measures ANOVA revealed a significant effect of flash number on total sleep for the 0–30 min window (*F*_(4,28)_ = 4.23; *p* < 0.0084).
(EPS)

S2 Fig Acute induction of sleep after stimulation with millisecond light flashes. (A) Representative EEG and EMG traces are shown after stimulation with 60 millisecond flashes administered every 2-sec for 2-min at ZT13. (B) Total sleep across ZT13 is shown in 15 minute bins. Two-way ANOVA (factors ‘light condition’ and ‘time’) with Bonferroni *post hoc* t tests were used to determine differences across time from flash onset. *p<0.05. Data represent mean ± SD.
(EPS)

S3 Fig Effects of lower ciproxifan doses on light-induced sleep. Mice (n = 4) were administered with saline and ciproxifan (3mg/kg and 6mg/kg) 20 min before a light pulse at ZT13. (A) Total sleep (% of saline) is shown. *ns*, Student’s paired *t* test (*p* = 0.31). (B,C) Delta/theta ratios of NREMS and wake during the light pulse were calculated and plotted (n = 3–4). Data represent mean ± SD.
(EPS)

## References

[1] Morin LP, Allen CN. The circadian visual system, 2005. Brain Res Rev. 2006;51: 1–60. 16337005 10.1016/j.brainresrev.2005.08.003

[2] Borbely AA, Huston JP, Waser PG. Control of sleep states in the rat by short light-dark cycles. Brain Res. 1975;95: 89–101. 168939 10.1016/0006-8993(75)90209-7

[3] Muindi F, Zeitzer JM, Colas D, Heller HC. The acute effects of light on murine sleep during the dark phase: Importance of melanopsin for maintenance of light-induced sleep. Eur J Neurosci. 2013. 10.1111/ejn.12189 PMC5079684 23510299

[4] Alfoldi P, Franken P, Tobler I, Borbely AA. Short light-dark cycles influence sleep stages and EEG power spectra in the rat. Behav Brain Res. 1991;43: 125–131. 1867754 10.1016/s0166-4328(05)80062-2

[5] Rusak B. Neural mechanisms for entrainment and generation of mammalian circadian rhythms. Fed Proc. 1979;38: 2589–2595. 499575

[6] Panda S, Provencio I, Tu DC, Pires SS, Rollag MD, Castrucci AM, et al Melanopsin is required for non-image-forming photic responses in blind mice. Science. 2003;301: 525–527. 12829787 10.1126/science.1086179

[7] Lucas RJ, Douglas RH, Foster RG. Characterization of an ocular photopigment capable of driving pupillary constriction in mice. Nat Neurosci. 2001;4: 621–626. 11369943 10.1038/88443

[8] Ruby NF, Brennan TJ, Xie X, Cao V, Franken P, Heller HC, et al Role of melanopsin in circadian responses to light. Science. 2002;298: 2211–2213. 12481140 10.1126/science.1076701

[9] Tsai JW, Hannibal J, Hagiwara G, Colas D, Ruppert E, Ruby NF, et al Melanopsin as a sleep modulator: Circadian gating of the direct effects of light on sleep and altered sleep homeostasis in Opn4(-/-) mice. PLoS Biol. 2009;7: e1000125 10.1371/journal.pbio.1000125 19513122 PMC2688840

[10] Mrosovsky N, Hattar S. Impaired masking responses to light in melanopsin-knockout mice. Chronobiol Int. 2003;20: 989–999. 14680139 10.1081/cbi-120026043

[11] Lupi D, Oster H, Thompson S, Foster RG. The acute light-induction of sleep is mediated by OPN4-based photoreception. Nat Neurosci. 2008;11: 1068–1073. 10.1038/nn.2179 19160505

[12] Altimus CM, Guler AD, Villa KL, McNeill DS, Legates TA, Hattar S. Rods-cones and melanopsin detect light and dark to modulate sleep independent of image formation. Proc Natl Acad Sci U S A. 2008;105: 19998–20003. 10.1073/pnas.0808312105 19060203 PMC2596746

[13] van Oosterhout F, Fisher SP, van Diepen HC, Watson TS, Houben T, VanderLeest HT, et al Ultraviolet light provides a major input to non-image-forming light detection in mice. Curr Biol. 2012;22: 1397–1402. 10.1016/j.cub.2012.05.032 22771039 PMC3414846

[14] Morin LP, Lituma PJ, Studholme KM. Two components of nocturnal locomotor suppression by light. J Biol Rhythms. 2010;25: 197–207. 10.1177/0748730410369890 20484691 PMC3063651

[15] Van Den Pol AN, Cao V, Heller HC. Circadian system of mice integrates brief light stimuli. Am J Physiol. 1998;275: R654–7. 9688706 10.1152/ajpregu.1998.275.2.R654

[16] Nelson DE, Takahashi JS. Sensitivity and integration in a visual pathway for circadian entrainment in the hamster (mesocricetus auratus). J Physiol. 1991;439: 115–145. 1895235 10.1113/jphysiol.1991.sp018660 PMC1180102

[17] Vidal L, Morin LP. Absence of normal photic integration in the circadian visual system: Response to millisecond light flashes. J Neurosci. 2007;27: 3375–3382. 17392453 10.1523/JNEUROSCI.5496-06.2007 PMC2568885

[18] Studholme KM, Gompf HS, Morin LP. Brief light stimulation during the mouse nocturnal activity phase simultaneously induces a decline in core temperature and locomotor activity followed by sleep. Am J Physiol Regul Integr Comp Physiol. 2013;304: 459–471. 10.1152/ajpregu.00460.2012 PMC3602824 23364525

[19] Morin LP, Studholme KM. Millisecond light pulses make mice stop running, then display prolonged sleep-like behavior in the absence of light. J Biol Rhythms. 2009;24: 497–508. 10.1177/0748730409349059 19926809 PMC2853800

[20] Morin LP, Studholme KM. Separation of function for classical and ganglion cell photoreceptors with respect to circadian rhythm entrainment and induction of photosomnolence. Neuroscience. 2011;199: 213–224. 10.1016/j.neuroscience.2011.09.057 21985934 PMC3237860

[21] Hattar S, Kumar M, Park A, Tong P, Tung J, Yau KW, et al Central projections of melanopsin-expressing retinal ganglion cells in the mouse. J Comp Neurol. 2006;497: 326–349. 16736474 10.1002/cne.20970 PMC2885916

[22] Muindi F, Zeitzer JM, Heller HC. Retino-hypothalamic regulation of light-induced murine sleep. Front Syst Neurosci. 2014;8: 135 10.3389/fnsys.2014.00135 25140132 PMC4121530

[23] Hubbard J, Ruppert E, Gropp CM, Bourgin P. Non-circadian direct effects of light on sleep and alertness: Lessons from transgenic mouse models. Sleep Med Rev. 2013. 10.1016/j.smrv.2012.12.004 23602126

[24] Panula P, Kaartinen M, Macklin M, Costa E. Histamine-containing peripheral neuronal and endocrine systems. J Histochem Cytochem. 1985;33: 933–941. 3894504 10.1177/33.9.3894504

[25] Watanabe T, Taguchi Y, Hayashi H, Tanaka J, Shiosaka S, Tohyama M, et al Evidence for the presence of a histaminergic neuron system in the rat brain: An immunohistochemical analysis. Neurosci Lett. 1983;39: 249–254. 6355911 10.1016/0304-3940(83)90308-7

[26] Watanabe T, Taguchi Y, Shiosaka S, Tanaka J, Kubota H, Terano Y, et al Distribution of the histaminergic neuron system in the central nervous system of rats; a fluorescent immunohistochemical analysis with histidine decarboxylase as a marker. Brain Res. 1984;295: 13–25. 6713171 10.1016/0006-8993(84)90811-4

[27] Takahashi K, Lin JS, Sakai K. Neuronal activity of histaminergic tuberomammillary neurons during wake-sleep states in the mouse. J Neurosci. 2006;26: 10292–10298. 17021184 10.1523/JNEUROSCI.2341-06.2006 PMC6674640

[28] Saper CB, Fuller PM, Pedersen NP, Lu J, Scammell TE. Sleep state switching. Neuron. 2010;68: 1023–1042. 10.1016/j.neuron.2010.11.032 21172606 PMC3026325

[29] Arrang JM, Garbarg M, Schwartz JC. Autoinhibition of histamine synthesis mediated by presynaptic H3-receptors. Neuroscience. 1987;23: 149–157. 2446202 10.1016/0306-4522(87)90279-x

[30] Arrang JM, Garbarg M, Schwartz JC. Auto-inhibition of brain histamine release mediated by a novel class (H3) of histamine receptor. Nature. 1983;302: 832–837. 6188956 10.1038/302832a0

[31] Arrang JM, Garbarg M, Schwartz JC. Autoregulation of histamine release in brain by presynaptic H3-receptors. Neuroscience. 1985;15: 553–562. 4022339 10.1016/0306-4522(85)90233-7

[32] Schwartz JC, Arrang JM, Garbarg M, Pollard H, Ruat M. Histaminergic transmission in the mammalian brain. Physiol Rev. 1991;71: 1–51. 1846044 10.1152/physrev.1991.71.1.1

[33] Itoh Y, Oishi R, Nishibori M, Saeki K. Characterization of histamine release from the rat hypothalamus as measured by in vivo microdialysis. J Neurochem. 1991;56: 769–774. 1704419 10.1111/j.1471-4159.1991.tb01990.x

[34] Monti JM, Jantos H, Boussard M, Altier H, Orellana C, Olivera S. Effects of selective activation or blockade of the histamine H3 receptor on sleep and wakefulness. Eur J Pharmacol. 1991;205: 283–287. 1667912 10.1016/0014-2999(91)90911-9

[35] Griebel G, Decobert M, Jacquet A, Beeske S. Awakening properties of newly discovered highly selective H(3) receptor antagonists in rats. Behav Brain Res. 2012;232: 416–420. 10.1016/j.bbr.2012.04.033 22561131

[36] Lin JS, Sakai K, Vanni-Mercier G, Arrang JM, Garbarg M, Schwartz JC, et al Involvement of histaminergic neurons in arousal mechanisms demonstrated with H3-receptor ligands in the cat. Brain Res. 1990;523: 325–330. 2169324 10.1016/0006-8993(90)91508-e

[37] Parmentier R, Anaclet C, Guhennec C, Brousseau E, Bricout D, Giboulot T, et al The brain H3-receptor as a novel therapeutic target for vigilance and sleep-wake disorders. Biochem Pharmacol. 2007;73: 1157–1171. 17288995 10.1016/j.bcp.2007.01.002

[38] Kathmann M, Schlicker E, Marr I, Werthwein S, Stark H, Schunack W. Ciproxifan and chemically related compounds are highly potent and selective histamine H3-receptor antagonists. Naunyn Schmiedebergs Arch Pharmacol. 1998;358: 623–627. 9879720 10.1007/pl00005303

[39] Colas D, Valletta JS, Takimoto-Kimura R, Nishino S, Fujiki N, Mobley WC, et al Sleep and EEG features in genetic models of down syndrome. Neurobiol Dis. 2008;30: 1–7. 10.1016/j.nbd.2007.07.014 18282758 PMC4689324

[40] Schmidt TM, Do MT, Dacey D, Lucas R, Hattar S, Matynia A. Melanopsin-positive intrinsically photosensitive retinal ganglion cells: From form to function. J Neurosci. 2011;31: 16094–16101. 10.1523/JNEUROSCI.4132-11.2011 22072661 PMC3267581

[41] Esbenshade TA, Browman KE, Bitner RS, Strakhova M, Cowart MD, Brioni JD. The histamine H3 receptor: An attractive target for the treatment of cognitive disorders. Br J Pharmacol. 2008;154: 1166–1181. 10.1038/bjp.2008.147 18469850 PMC2483387

[42] Toyota H, Dugovic C, Koehl M, Laposky AD, Weber C, Ngo K, et al Behavioral characterization of mice lacking histamine H(3) receptors. Mol Pharmacol. 2002;62: 389–397. 12130692 10.1124/mol.62.2.389

[43] Gondard E, Anaclet C, Akaoka H, Guo RX, Zhang M, Buda C, et al Enhanced histaminergic neurotransmission and sleep-wake alterations, a study in histamine H3-receptor knock-out mice. Neuropsychopharmacology. 2013. 10.1038/npp.2012.266 PMC3629391 23303066

[44] Lin JS. Brain structures and mechanisms involved in the control of cortical activation and wakefulness, with emphasis on the posterior hypothalamus and histaminergic neurons. Sleep Med Rev. 2000;4: 471–503. 17210278 10.1053/smrv.2000.0116

